# The refugee waves and the continuum of violence experienced by Ukrainian refugee women in Bulgaria

**DOI:** 10.3389/fsoc.2025.1587585

**Published:** 2025-07-15

**Authors:** Alexey Pamporov

**Affiliations:** Institute of Philosophy and Sociology at the Bulgarian Academy of Sciences, Sofia, Bulgaria

**Keywords:** Ukrainian refugees, temporary protection, continuum of violence, gender based violence, VAWG, GBV

## Abstract

This article examines the continuum of violence experienced by Ukrainian refugee women in Bulgaria over the past 3 years, following the full-scale invasion by the Russian army. The study draws on a secondary analysis and triangulation of three quantitative surveys commissioned by UNHCR and UNICEF in Bulgaria, along with three waves of a randomized socio-economic survey funded by UNHCR. Employing a constructivist grounded theory approach, the article proposes a typology of several refugee waves. It argues that the period of arrival, the means of arrival, and the type of accommodation selected reflect the survival strategies of refugee women and may influence their exposure to both community-based and transnational gender-based violence (GBV). The findings indicate that certain institutional features of Bulgaria’s state accommodation programme for individuals with temporary protection status expose women and girls to additional risks of GBV, including survival sex and relocation to areas associated with commercial sex work. The vulnerability of Ukrainian refugees is further exacerbated by three country-specific factors: institutional neglect of violence against women and girls; widespread acceptance of cultural myths related to sexual violence; and prevailing national stereotypes targeting Ukrainians and, more broadly, women from certain Slavic backgrounds. The analysis clarifies the various forms of the continuum of violence affecting the Ukrainian women and girls in Bulgaria and confirms the patterns of “slow violence” and the “violence of uncertainty,” observed in the Eastern Mediterranean region by other international studies.

## 1 Introduction and theoretical framework

The full-scale invasion of the Russian army in Ukraine after February 24, 2022 has triggered an unprecedented influx of people seeking international protection in Bulgaria.^1^ From the first day of the military raid until December 31, 2024, there were about 3,577,870 “arrivals,” i.e., various Ukrainian citizens crossed the Bulgarian border (some of them more than once in the period), according to the Bulgarian Border police data, published by UNHCR. Out of this figure, about 203,122 persons were granted temporary protection status under the European Temporary Protection (Decision (EU) 2022/382), of which 74,739 remained living in Bulgaria by January 15, 2025. The proportion of adult women is 49% and the proportion of children is 30% (of which a bit more than a half are girls), i.e., the women and girls currently living in Bulgaria are approximately 65% of the Ukrainian refugees in Bulgaria.^2^ The first MSNA study shows that in the Ukrainian refugee population in Bulgaria there are 28% of single mothers and almost 22% of adult single women, without children (UNHCR, 2023), and the follow up SEIS study show that about 62% of the households are headed by woman (UNHCR, 2025). However, the latter study indicates a significant increase in the female experience with verbal harassment from 37% in 2023 to 45% in 2024, but being threatened with violence decreased from 17% in 2023 to 11.7% in 2024 (UNHCR, 2025).

Already in the early 1990s, some authors argued that violence against women is not haphazard but systemic, reflecting deep-rooted gender inequalities (Jeffreys, 1990) and sexual violence in particular was regarded is a political act, ingrained in patriarchal structures that reinforce male dominance (MacKinnon, 1989). In line with that, the violence against women was further regarded not as an anomaly or personal tragedy, but as one of the six foundational structures of patriarchy (alongside labor market, family life, national cultures, sexuality, and the public institutions). The concept of “gender regimes” in that line was introduced as a specific set of gender interactions within private patriarchy in the domestic sphere and institutional patriarchy centered around the public policies in educational, healthcare and labor structures (Walby, 1990). A milestone of these efforts and problematizations was the United Nations Declaration on the Elimination of Violence against Women, which for the first time offered a formal, international definition of violence against women as “any act of gender-based violence that results in, or is likely to result in, physical, sexual or psychological harm or suffering to women” (UN General Assembly, 1993, Article 1). The declaration framed GBV not only as a private issue but as a matter of human rights and international concern, urging states to adopt legislation, preventive measures, and institutional responses. Despite that, some contemporary studies show that not only totalitarian and theocratic, but also various neoliberal policies and in general the global militarization aggravate women’s exposure to violence. And that this occurs not only in war crises but particularly in post-conflict and resource-poor contexts (Hester, 2012; True, 2012). The persistent global prevalence of GBV, despite legal reforms and human right awareness campaigns, illustrates the depth of its structural roots (Reilly, 2019). In the context of war and displacement, GBV becomes even more pronounced. Following the Russian invasion in 2022, there has been a documented rise in sexual violence both in war zones and among Ukrainian refugee diasporas in host countries across EU. The UN mission in Ukraine reported on conflict-related sexual violence occurring between January and December 2022, documenting incidents and emphasizing the need for strategic actions to address such violations (UN HRMMU, 2023). The UN Population Fund has also highlighted that the ongoing war amplifies the vulnerabilities of women and girls, increasing the risks of gender-based violence, sexual exploitation, and abuse. Displacement and the destruction of public infrastructure have shrunk the access to support services bellow the critical levels many females, including both regular health care facilities and specialized psychological support for survivors of violence (UNFPA, 2025).

Having in mind the observed and reported different forms of violence, ranging from culturally normalized gender-based harassment to severe and criminalized sexual assault, the current article finds out as even more suitable to the current context the use of the concept “continuum of violence”—as an important tool to understand and challenge gender-based violence (GBV) as a routine misogyny and sexism (Kelly, 1988). It fits properly the situation of the post-Soviet countries and the series of armed border conflicts after the collapse of the USSR, for example: Transnistria, Abkhazia, South Ossetia, Crimea, etc. Those cases doubtlessly confirm the postulation that the boundaries between wartime and peacetime violence are fluid, especially for women, for whom violence does not necessarily begin or end with formal armed conflict (Cockburn, 2004), since the violence against women and girls could be perpetuated both by militarized structures or by civilian law enforcement agencies: soldiers, aid workers, border officials and police officers (Enloe, 2000). The assumption of the current article is that experiences of the Ukrainian women and girls, with temporary protection status in EU, do not differ significantly from the already observed experiences of migrant and refugee women, particularly in contexts of forced displacement. Therefore, one may expect that GBV is persistent and interconnected, rather than episodic and should not be regarded as isolated acts but occurs across a temporal and spatial continuum, affecting women before, during, and after migration (Tastsoglou, 2022). Moreover, fleeing the Donetsk, Luhansk, Zaporizhian and Odessa regions, and using mainly Russian language in their everyday life, the Ukrainian refugees in Bulgaria are additionally exposed on the intersectionality risks of overlapping hatred: xenophobia, misogyny and classism (Crenshaw, 1989). In other words, it may be expected that they are systematically marginalized and exposed on structural inequalities, based on multiple traits: migration status, economic precarity, cultural background and gender (Collins, 2000). Moreover, a recent study showed that the original theory proposed by Kelly needs to be modified to accommodate the very unique forms of GBV that refugee women experience - forms that are different than those affecting the general population of women: “slow violence,” “cultural shaming,” “economic coercion,” and at least but not at last the “continuum of precarity,” regarded as refugee specific intersectionality due to overlapping between legal, structural, cultural, and interpersonal violences during migration (Sisic et al., 2024). Several studies underline also a third dimension—a socially induced vulnerability of Ukrainian women—due to the inaction of the state and all prejudices and negative gender-based stereotypes (Zinkevych et al., 2024), they are more likely to report only economic violence (Capasso et al., 2022), but “violence and precarity are co-constituted” (Tastsoglou et al., 2021, p. 1).

## 2 Data and methods

The current article relies on secondary analysis of data, gathered through three qualitative studies, conducted by the author for UNHCR and UNICEF in Bulgaria and three quantitative randomized surveys conducted by Global Metrix Ltd. for UNHCR in the period 2022–2024, in which the author was a key methodological expert in charge of sampling model, cultural adjustment of the standardized UNHCR set of questions, as well as for the sensitivity training of the enumerators.

**Figure 1:**
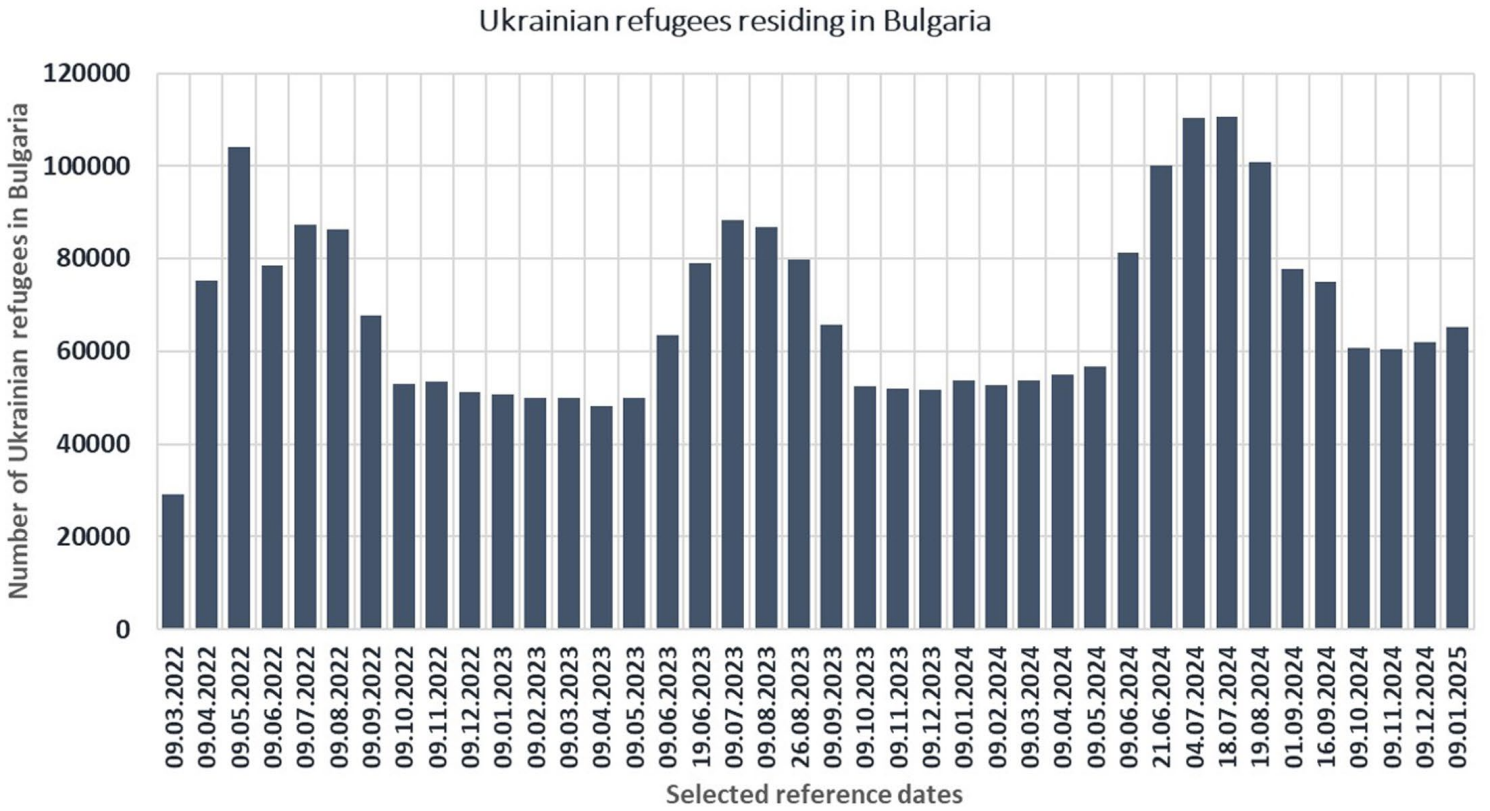
Number of Ukrainian refugees present in Bulgaria by selected date (arrivals minus departures) in the period March 2022–January 2025. Source: UNHCR.

Using the grounded theory approach of axial coding (Strauss and Corbin, 1990) and data triangulation of six different studies, the current article aims to explain the different migration patterns of the refugees and to illustrate how these patterns impact the continuum of gender-based violence experienced by some refugee women in Bulgaria. The idea about the probable importance of the arrivals and departures of the refugees came from the “waves-like” general migration pattern of the Ukrainian citizens with temporary protection status in Bulgaria (illustrated at Figure 1), but meanings were constructed (Charmaz, 2008) in the interactions of the author with Ukrainian refugees in various locations in the six NUTS-3 regions of Bulgaria, with the highest number of resettled Ukrainians. One might critique the assumption that a given social phenomenon exists, arguing that such an assumption can lead to overinterpretation (Eco et al., 1992). In this view, the perceived significance of the “waves” may reflect a self-fulfilling expectation, resulting in an illusory correlation with gender-based violence (GBV). However, this article adopts a hermeneutic perspective, which acknowledges that analysis always begins with certain preconceptions. These preconceptions are not fixed but are subject to critical reflection and can be revised in pursuit of a more adequate and accurate understanding of the phenomenon under study (Gadamer, 2004).

### 2.1 Qualitative studies

The first qualitative study was set as a rapid assessment procedure (Harris et al., 1997) by UNHCR in early September 2022, as a response to the increasing needs of the Ukrainian refugees and with regard to the forthcoming winter. The methodology of the study was typical of “an urgent anthropology” fieldwork (Zhelyazkova, 2003) and initial mapping of the situation, based on direct observations and focused group discussions (Khan and Manderson, 1992). The study started with the “tabula rasa” presumption, i.e., we know nothing about the emerging situation and there was not strict academic research question and hypotheses. It was rather an applied and descriptive first step in a “Theory-of-change” to be, aiming at initial mapping of the refugees’ background and needs: Places and regions of origin within Ukraine; Means of transportation and route; Household composition in Bulgaria and information for the people left behind; Educational and Labor backgrounds; Healthcare status and knowledge for the healthcare access in Bulgaria. The qualitative sampling was done in a multiple case manner (Miles and Huberman, 1994) in five locations: Varna, Golden Sands resort, Burgas, Stara Zagora and Sofia. The data included the refugee stories and attitudes of 52 persons.

The second qualitative study followed the same multiple-case sampling model and relied on 6 focused group discussions, involving 45 women, mothers of children aged 0–7 years. The study was conducted in late March—early April 2023 in Burgas, Pomorie,^3^ Sofia (2 groups) and Golden Sands resort. The study showed that after a year of stay in Bulgaria under the temporary protection, the displaced persons from Ukraine, are still facing various structural barriers in their access to health care and early childhood education, but those barriers are hindering Bulgarian citizens in the same way: the unequal distribution of hospital and pre-hospital care in Bulgaria, and lack of places in the nurseries and kindergarten in the metropolitan cities. It was commissioned and funded by UNICEF Bulgaria.

The third study was a Stock taking exercise on the educational support for Ukrainian refugee children in Bulgaria, also commissioned and funded by UNICEF Bulgaria and conducted in April–October 2024. Due to the specifics of the study, a judgmental sampling based on stakeholder analysis was used, covering the project activities of 9 non-government organizations as follows (in alphabetical order): ADRA Bulgaria, Dokova and Dokov for future foundation, FAR foundation, For our children foundation, Fund “GOOD,” Personal development support center—Burgas, Situational center “Open doors,” Ukraine support and renovation (“Second home”) and Ukrainian hive (“Vulyk”). The study included opinions of 69 persons, of which were 25 children. The children reported rather high levels of school bullying and low discipline in the Bulgarian system but this type of violence remained out of the scope of the current article.

### 2.2 Quantitative studies

The UNHCR commissioned three waves of randomized surveys among Ukrainian refugees in Bulgaria. The first leg, labeled “Winter needs” was a standardized Multi-Sector Needs Assessment (MSNA) based on four independent randomized samples, related to public policies aimed at four target and partially overlapping populations: Accommodated at state facilities; Parents of children enrolled in the Bulgarian educational system; New arrivals, users of Blue Dots;^4^ and Ukrainians living at free addresses. It was conducted in December 2022–January 2023 with total sample size *N* = 1,311 respondents. The outcomes of the first leg were published in 2023 as a separate report (UNHCR, 2023).

Based on the lessons learned from the first survey, the second and third legs were conducted in July–August 2023 (*N* = 1,054 households) and June–July 2024 (*N* = 1,072 households), in a two sub-sample mode: refugees in state accommodations and refugees in private accommodations. The study was rebranded as Socio-Economic Insights Survey (SEIS). A preliminary comparative study between the second and third waves (i.e., August 2023 and July 2024) was published in the beginning of 2025 (UNHCR, 2025). Due to the different timing of the first wave (January 2023), different sampling strategy and partially modified questionnaire, the comparison between the first and other legs is not always possible.

## 3 Research outcomes

### 3.1 Migration patterns

#### 3.1.1 The summer vacations: a spurious correlation

The official data about the arrivals and departures of the Ukrainian refugees in Bulgaria, published online by UNHCR, at first glance confirms one of the broadly expressed negative stereotypes towards the Ukrainian refugees in Bulgaria: They are coming here not because really need to escape the war but because want to use the benefits of free accommodation on the Bulgarian seaside during the summer.^5^ The peak of their presence is from June to August in each of the three war years and in the autumn their number declines. However, it turned out that this is a spurious correlation because the statistical trend relates not to the summer holidays but it is a due to the rhythm of the war and airstrikes against Odessa and artillery attacks against Dnipro and Kharkiv regions, as far as there is rather accurate data available online.^6^ Of course, the intervals correlate also with summer days of in the Ukrainian school system and in general with the summer holidays of the working people but the real push factor is the intensity of the conflict in the summer, the holidays just make it easy for the children and their working mothers to live the country and “commute” to Bulgaria for the season.

At the same time, the triangulation of the qualitative data makes it possible to distinguish four “ideal types” or four “waves” of migrant patterns of the arrivals in Bulgaria, with clear relation to the period of arrival, transportation means, the route, the push and pull factors, as well as in the barriers to integration. Of course, as can be seen from the statistics above, the Ukrainian refugees entering Bulgaria are hundreds of thousands and therefore it is possible to have mixed types and interweaving between the particular cases, as well as it is possible to have other types of migratory behavior resulting from the characteristics of those who have used Bulgaria only as a transit country and who have not settled permanently. The proposed typologies below are “ideal” in a Weber (1949) sense, i.e., they are researcher’s constructs, which enrich the comparison; enhance the subjective meanings that individuals attach to their actions; and assist in identifying causal relationships and historical variations. Hence, they allow us to pay attention to the need for specific public policies and specific forms of support for different Ukrainian citizens in the context of gender-based violence and risk assessment of the actual continuum of violence.

#### 3.1.2 The first wave: *“the hen is not a bird, and Bulgaria is not abroad”*^7^

The Ukrainian refugees of the first wave came mainly from Kiev, Kharkiv, Lugansk, Donetsk and Kherson. These were the areas initially affected by the “military operation” announced by the Russian Federation. The stories that people from this wave tell are stories related to the imminent danger to their lives. There is no preliminary plan or chosen destination, and decisions are made on the run: *“In half an hour we packed two bags and set off”* (female, 28). For this reason, the individual routes showed the following model of wandering: “Kiev → Odessa → Moldova → Varna”; “Orlovka → Isaccea → Balchik → Svilengrad → Sofia”; “Odessa → Poland → Moldova → Sofia”; “Mikolaiv → Tvarditsa → Stara Zagora.”

The main part of this wave was composed mostly by mothers with one or two children, as well as by elderly couples (some of whom were on holiday in Bulgaria before 1989). Unplanned travel leads to the fact that there is no common transportation pattern and moreover various stories were told about crossing the border with Romania on foot, about travelling by ferry, by train and by private cars. Thinking through the pyramid of needs (Maslow, 1943), the main push factor was the need for safety, driven by the imminent attacks on the Ukrainian homes. The driving needs in this case are the protection of children: *“We are here only for the children”* (female, 41) and the serenity of older people. In some cases, middle-aged mothers come with their elderly parents in order to have someone to help them with child care. In other cases, the elderly have some chronic illness and also need care. Thus, some middle-aged women have a triple burden: finding work in Bulgaria, taking care of their children, and taking care of the elderly parents who have accompanied them.

There are two main pull factors of the first wave: *“Bulgaria is close to Ukraine and the language is very similar and easy to learn”* (female, 44). The first factor—Cyrillic script—is perceived as a great advantage in terms of quick integration of children and elderly who do not know another language well. For example, the woman quoted in the previous sentence lives in Bulgaria with her second and third children (aged 8 and 9), while her eldest daughter is 27 years old and lives in Italy. The second factor is the proximity to Ukraine. It is related to the initial expectations of both the international community and the Ukrainian refugees themselves that this is a “military operation,” i.e., that the armed conflict will end within a few months and then it is better to be close for them in order to get home more easily. Some of the routes mentioned above are in fact the routes of regular international bus services between Bulgaria and Ukraine. The first wave of Ukrainian refugees managed to benefit from civic initiatives and voluntary support of ordinary Bulgarian citizens. Some of the people from this wave received international protection in the form of humanitarian status even before the activation of the temporary protection mechanism. From the very beginning they have been accommodated in private accommodations, for which in some cases they did not pay any rent. They have private doctors. The children have been enrolled in kindergartens and schools since the second term of the school year 2021/2022. In terms of time, it can be said that the first wave started on February 24, 2022 and covered cases until March 15, 2022, shortly after the activation of EU Council Directive 2001/55/EC (2025) on minimum standards for the granting of temporary protection, or until 8 April, when, according to Order RD05-263, the initiation of proceedings of Ukrainian nationals for international protection was prohibited and suspended.

The integration of the Ukrainians of the first wave was hindered by the uncertainty of the support provided by the Bulgarian authorities. The Government was constantly changing the parameters of the state support—in terms of funding and conditions, on the one hand, and on the other hand, it set very short horizons of support: the end of May 2022, the beginning of October 2022, the end of February 2023, etc. This created a paradox, where Ukrainian refugees came because of the need for security, but public policies in Bulgaria set up such a process of “integration” that brought uncertainty.

The second obstacle towards integration was the shortage of certified Bulgarian language courses, which is a condition for inclusion in the labor market for some of the highly skilled professions (e.g., doctors). Moreover, most of the certified training institutions are used to working with students, i.e., young adults. However, there was a need for a rapid development of curricula tailored to the different age levels and relevant needs and skills in training children, as well as in developing specialized programmes for persons in the third age.

The third barrier is rooted in structural gender inequalities within the labor market, a pattern observable not only in Bulgaria, but across Europe and present in Ukraine as well. These inequalities are reflected in the feminization of certain professions, often limiting women’s carriers and access to broader employment opportunities. In the initial months of 2022, women made up 77.4% of the adult Ukrainian refugees granted temporary protection in Bulgaria. Consequently, there was a significant overrepresentation of highly qualified professionals—such as accountants, tax inspectors, and legal advisors—whose expertise is specific to Ukrainian legal and institutional frameworks and thus not easily transferable. Similarly, professions like civil servants, teachers, and pedagogical counselors, which require advanced language skills and cultural familiarity, faced limited opportunities for adequate employment. This situation highlights a pronounced mismatch between refugees’ qualifications and the demands of the Bulgarian labor market, exacerbated by systemic barriers related to both gender and professional specialization.

#### 3.1.3 The second wave: “golden sands and sunny beach”^8^

The Ukrainians who came with the second wave are from all over Ukraine and the refugees from the initially affected areas were joined by residents of Zhytomyr, Vinnitsa, Ternopil and Chernivtsi. The start of this wave was tentatively April 1, 2022—when the state formally started paying to the private accommodation establishments^9^ and this encouraged a significant part of the tourism industry to join in and open their doors “out of a season.” However, the second wave was rather linked to the escalation of the conflict in Ukraine after April 8, 2022 and the introduction of the EU’s fifth package of restrictive measures against Russia, following revelations of multiple civilian casualties. Although the Ukrainian President’s Decree 64/24.02.2022 provides, as an exception, that men in large families and men who are single parents will be able to leave the country despite the war situation, many families initially did not take this opportunity because they were uncertain whether the border authorities will still allow them and because they did not know where they could go with their many children. Thus, in the second wave, a significant proportion are large families and extended family households with grandparents over 60 years of age. In contrast to the chaotic route of the first wave, the second wave came with clear transport trajectories, of which the most recurrent ones coincide with the routes of Bulgarian and Ukrainian tourist transports: “Odessa → Varna”; “Bolhrad → Varna”; “Kiev → Odessa → Ismail → Varna → Sofia.” Of course, the main push factor was the expansion of the military conflict and the end of the illusion that this is an “operation” and will end quickly. Another factor, however, is the possibility that, in addition to security needs, the social needs of belonging and living in a community could be met. Hotel accommodation is in fact accelerating the creation of nests of Ukrainian diasporas where children and adults feel *“among their own kind”* (male, 63).

Admittedly, the major pull factor in this regard was the hotel accommodation programme. On the one hand, it was encouraged by the positive memories of Bulgarian Black Sea resorts—dating back to before 1989—and, on the other hand, by the guaranteed satisfaction of the need for security: a clear route of travel, a clear pattern of accommodation, guaranteed shelter and food for the children and the household: *“You get on the bus and you arrive in one piece. In Germany, they kidnap children from trains”* (female, 33).

Although hotel accommodation in resorts was considered a great advantage and “luxury” in the treatment of Ukrainian refugees, it turned out that it was also the biggest barrier to their integration in Bulgaria. On the one hand, dozens of “Little Ukraines” have been created in resort towns along the Black Sea coast, which removed the need for children and adults to come into contact with Bulgarian citizens. Thus, a process of hidden ghettoization and encapsulation within one’s own community was observed. On the other hand, the resorts are places of rest and entertainment—there are bars, restaurants, casinos, etc., but there is a lack of access to basic services related to normal everyday life: there are no schools, no hospitals and no adequate pre-hospital care, pharmacies are not open out of the season, and there are not enough jobs outside the hotel accommodation and restaurant sector (which also does not offer full employment out of the season).

After May 31, 2022, the Bulgarian government changed the accommodation programme and from 40 BGN per person per day, the subsidy became 15 BGN. Thus, many hotels refused to accept Ukrainian refugees and they were asked to leave their respective places. With the start of the tourist season and the possibility for hotels to make much larger profits on the free tourist market, there was even more pressure on refugees. Conventionally, the end of the second wave can be put at June 30, 2022, when the momentum of organized Ukrainian arrivals through tour companies disappeared (since the companies shifted back to usual TUI costumers). The statistics available to the Bulgarian UNHCR office, provided by the DG Border Police, confirm the conditional limits of this wave.

#### 3.1.4 The third wave: “it is cheaper here”

The third wave started tentatively around June 1, 2022—when it was clear that there would rather no longer be available accommodation in hotels under the government program, and the main peak of the wave was after mid-August. The wave was, of course, associated with another escalation of the war conflict. On the one hand, the Ukrainian army managed to take back some areas, but on the other hand, Russia changed command and announced partial mobilization. In most cases, Bulgaria was not the first choice for the respondents and they moved there after initial stay in another EU country and even from the USA. Some of the Ukrainian refugees in this wave are wives and children of men who work as sailors, or are wives of men who the war in Ukraine trapped as guestworkers in the EU. Through various mechanisms provided for in Decree 64/2022, middle-aged men have also been able to come to Bulgaria with this wave so as to be reunited with their families. The exception to the rest of the Ukrainian citizens in this wave are the Bessarabian and Taurian Bulgarians^10^ who come directly through the Romanian border near Izmail. The itineraries of the people in this wave are also very specific, with clear starting and ending points of the journey in advance. Some of the refugees even come with accommodation already found or work secured, via the internet. The great advantage of this wave is that they use the contacts and already established Ukrainian and Russian diasporas in Bulgaria.

The main push factor for the refugees of the third wave is the loss of hope that the war will end soon. The permanent shifts of the front line north or south and the gradual transfer of some areas from Russian to Ukrainian hands and vice versa led to the destruction of their homes, settlement infrastructure, and created extremely high insecurity for the lives of the civilian population. In this case, however, a key factor that builds on the needs for security and social interaction was the need for self-respect and self-improvement. The peak of the wave began in mid-August for a reason. The school year in Ukraine begins on the first of September and the families who decided to come with the third wave are families who wanted their children to have a “normal winter” (an expression by multiple respondents)—with warm and well-lit homes, in a “live-in” school system. The most striking in the choices of that wave, was the fact that even the planning of rental housing in many cases was tailored to the children’s extracurricular opportunities: ballet, piano lessons, access to sports and art schools. The pull factor in this case was the already well established Ukrainian and Russian diasporas—who provide ample information in advance about how a given family can arrange their life in Bulgaria. The second pull factor is that Bulgaria is a European Union country, which gives them a guarantee of peace and some quality of life, while at the same time the cost of living here is the lowest in the EU. This is why also a specific separation of families was observed: mothers with children reside in some of the big cities in Bulgaria, while their husbands (sailors and guest workers) send money transfers from abroad. Although they arrived the latest, people from this wave seem to be the most integrated. Most of them have already rented private accommodation or even bought a home. They have general practitioners. Children have been vaccinated according to the Bulgarian calendar and are enrolled in kindergarten or school, as well as in various extracurricular activities. Women and men of working age have found some work or are looking for alternative income and work options online. Additionally, they have relatives and friends in Bulgaria who came with the first wave.

#### 3.1.5 The fourth wave: “the seasonal commuters”

The second leg of SEIS study of UNHCR in Bulgaria, shows that about 87.5% of the Ukrainian refugee households are already living over a year in Bulgaria, and the average stay by July 2024 is about year and 11 months, i.e., most of the families have come with some of the first three waves. Moreover, 42% of the respondents did not consider the need to visit Ukraine, and 15 wanted but did not go, mainly because of security concerns. Approximately 5% of the total sample are currently so economically deprived that they are not able to cover the travel costs (UNHCR, 2025).

The triangulation with qualitative studies shows that those who stay permanently in Bulgaria are the one who have lost everything in Ukraine: no relatives and friends (due to war incidents or displacement), no occupation and no home (both due to total destruction of the industrial and residential infrastructures). They come mainly from Donetsk, Luhansk and Zaporizhian districts, most of which are under Russian control at the moment (mid-April 2025).

However, 29% of the refugees have been in Ukraine once and 14% have been in Ukraine more than once (UNHCR, 2025). The focused group discussions show that these people still have elder relatives and visit them, or are married women, visiting their husbands back at home in the military leaves (because the male are not allowed to travel abroad to prevent desertion). Some people travel to check if their home in Kyiv, Odessa or Kharkiv is still there or has been plundered by looters. Some travel because they successfully maintain some business relations. And last, but not least, some respondents have been back in Ukraine in order to look for return options: a new job or a new home in Kyiv, Lviv or western Ukraine in general, since they lost the faith that they will be able to return to the currently occupied territories. Therefore, the “fourth wave” is rather a metaphor, which overlaps and include arrivals from the first three waves, but depicts the status of seasonal “in-war commuters”: when the combat fights intense (in May–September), they stay in Bulgaria, but when they fell that it is rather safe in Ukraine—they travel back and contribute to the statistically observed waves, illustrated at Figure 1 (which gave the initial idea of this article).

#### 3.1.6 The “zero” wave: “the war didn’t start now”

**Figure 2:**
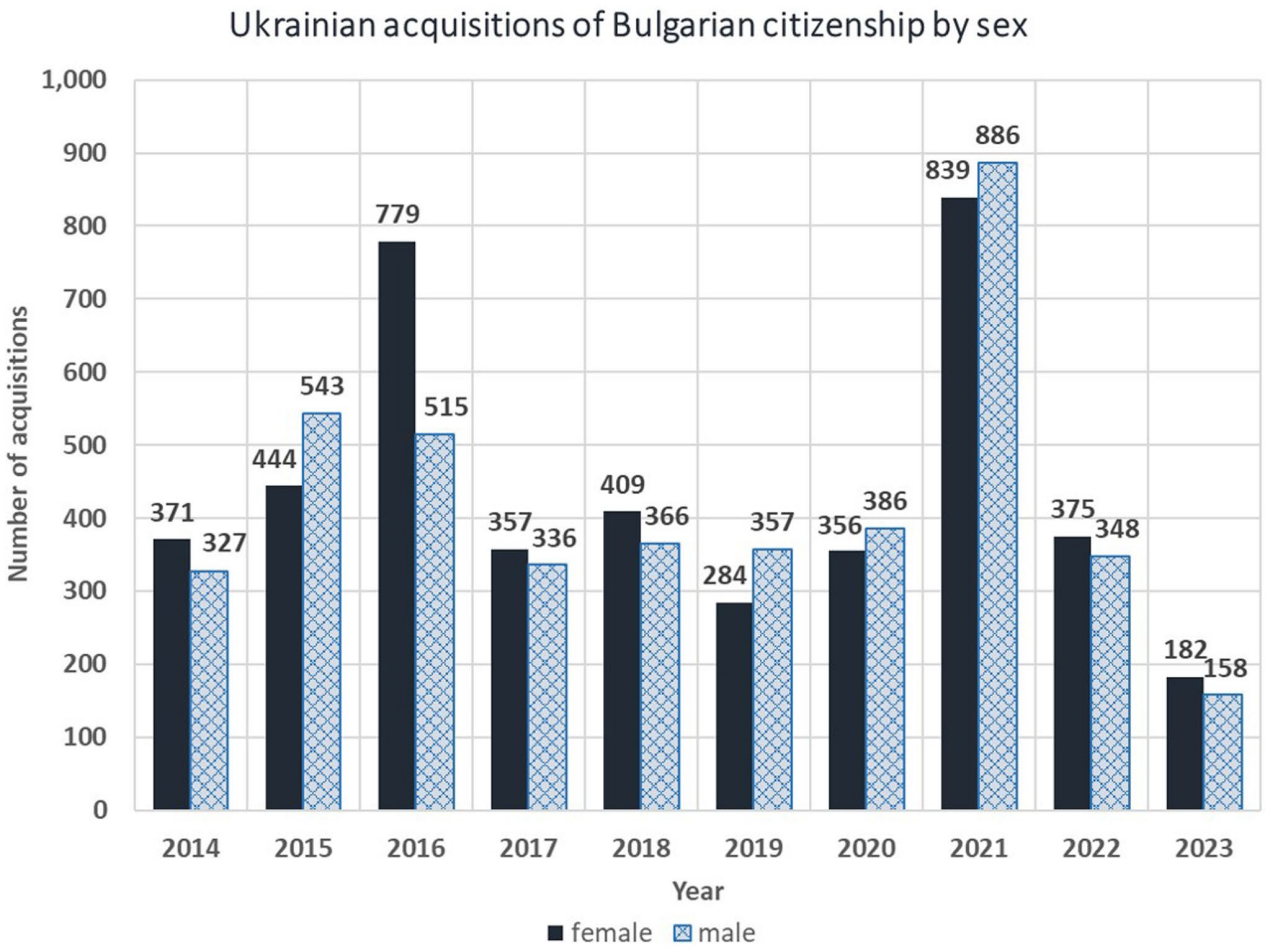
Acquisition of Bulgarian citizenship by sex, by former Ukrainian citizens in the period 2014–2023. Source: Eurostat (migr_acq__custom).

The interviews with respondents from the first and third waves, highlighted an important fact: *“The war did not start now, but 9 years ago”* (female, 48). There is a large, well-established Ukrainian diaspora of migrants who arrived between 2014 and 2021, mostly from the areas around Donetsk, Luhansk, Crimea and Odessa. These are men and women starting businesses as entrepreneurs, nuclear families with one or two children, and extended families of several generations. The reason for their migration is very well summed up in the statement of a Bessarabian Bulgarian woman who participated in one the discussions: *“We did not want to find ourselves suddenly in Russia”* (female, 43). In fact, the Ukrainian refugees of the zero wave are already well integrated and currently “invisible” in society. They are in private accommodations; they have private doctors; they have found jobs or started private businesses; they have PIN^11^ and a status other than temporary protection; their children are enrolled in Bulgarian schools. According to the Eurostat database (migr_asyappctza), in the period 2012–2023 there were 1,470 first time applications for asylum in Bulgaria by Ukrainian citizens, most of which (1285) in the first days of the Russian invasion in 2022. There are also 8,618 former Ukrainian citizens, who became Bulgarian citizens in the period 2014–2023. However, both figures do not include and consider the seasonal workers in the touristic sites, nor the businessmen with residence permits due to their established businesses. Moreover, in 2017 there was a media scandal in Bulgaria because of the discrepancy in the official figures published by Eurostat and official figures published by the Ministry of Justice, which are several times higher. The key issue in that case was the fact that the data, disseminated by Eurostat on citizenship acquisitions, are relevant only for persons who have a current address in Bulgaria, since the requirement of Regulation 862/2007 states: “Member States shall provide the Commission (Eurostat) with data on persons habitually residing on the territory of the country who have obtained citizenship of the country.” In other words, the Ukrainians with Bulgarian citizenship, who are back in Ukraine and have a permanent but not current addresses in Bulgaria, stayed invisible for the statistics. A data inquiry about the number of Ukrainian applications for Bulgarian citizenship shows two to three times higher totals in recent years: 1886 in 2020, 3,133 in 2021, 4,508 in 2022, and 3,141 in 2023, as illustrated on Figure 2.

### 3.2 The continuum of violence and the migration waves

Unfortunately, the situation of some of the invisible Ukrainian women from the zero wave, as well some women of the second wave are simply a textbook case for what is known as a continuum of violence against women in its whole spectrum: from misogynist jokes and sexual harassments to coerced sex (Kelly, 1988), and even sexual exploitation (Cockburn, 2004). The corpus of international publications clearly distinguishes four main domains of violence against women and girls: Violence in the family; Violence in the community; Violence perpetrated or condoned by the state; and Violence in the transnational sphere (Manjoo, 2012). With regard to the Ukrainian women in Bulgaria, at the moment there are two of the four main domains of violence observed in a very recognizable manner: violence in the community and transnational violence. Both are explicit cases of gender-based violence in migration: “rooted in gendered social norms and structures and unequal relations of power” (Freedman et al., 2023, p. 5), as described in the following paragraphs.

Already in 2016–2017 there were clear indicators for participation of Ukrainian and Moldavian women in commercial sex services (Petrunov, 2023) at the seaside resorts in Northern Bulgaria: Kavarna, Albena, Golden Sands and Varna; as well as in Sunny Beach in the South. Most of the ladies were recruited initially by job offers in tourism: reception desk staff, group guides and art animators, or SPA services. Soon after arrival the girls realized that the monthly wage of 500 BGN (approximately 250 EUR) was less than the regional average salaries at the time (approximately 350 EUR). With that money, the Ukrainian women were barely able to cover their own needs of food and dwelling, and, as a matter of fact that was just 10–15 EUR more than the average Ukrainian salary at the moment (something like 2/3 of the average monthly wage in Kyiv and 1.2 of the average monthly wage in Odessa). If one also includes the travel expenses, the working positions in Bulgaria were actually a loss instead of a profit. And this was the tricky point of the specific sexual exploitation: the Ukrainian ladies joined the VIP stratum of the commercial sex services: private parties and private sessions for rather exclusive (i.e., rich) clients. Moreover, it was set as a voluntary “double shift”: at the reception desk during the day, at the sex party during the evening or night. Formally, the Ukrainian females were not forced into commercial sex and it seemed to be their own choice but, de facto, it was survival sex, neglected by the Bulgarian anti-trafficking authorities. This pattern actually started around accession of Bulgaria in EU and uses the well-known blurred border between fashion modeling and commercial sex services at the “elite” stratum (Gounev et al., 2009).

The survival sex but in not so “fancy” manner reappeared in June 2022. After the end of the initial accommodation state programme, and with the start of the active tourist season, many of the Ukrainian refugees were threatened by eviction from their hotel accommodation. Without legal permanent income, without a place to live, accompanied by dependable minors, some of them have chosen to join commercial sex services. A series of current observations in Burgas, Varna, Plovdiv and Stara Zagora regions indicate that the recent pimps are not the hotel owners but some taxi drivers, which connect clients and girls and drive the girls to the clients. Of course, a disclaimer is needed here. This issue cannot be ascribed to all refugees accommodated at state premises and in private hotels but just to some of them; as well as it is observed among all migratory waves, too. Moreover, the higher income allows some of the refugee women to actually escape poverty and dependency on government support and to start another business, renting a dwelling or workspace on the free market. So, for some of them this was a means of survival and yet part of the continuum of violence, as it will be discussed further down.

## 4 Discussion

As it was stated already in the introduction, the secondary analysis of qualitative data carries a risk of overinterpretation in the generalization and in the conclusions. Therefore, it is important to keep in mind that the five waves outlined above are metaphors or “ideal” types, in order to highlight some underlying differences. At the same time, it should be stressed that refugees of all waves need security, social contacts, dignity and self-fulfillment. Refugees of all waves are people fleeing a war: *“We do not plan to go back to Ukraine because our home is ruined. We have nowhere to go back to”* (female, 39). Moreover, they need to culturally adjust to the host society but sometimes the political and social context of the host societies could be fueling the continuum of gender-based violence due to its own patterns of societal normalization of violence against women and girls and lack of sensitivity and awareness about the intersectional vulnerability of the refugees by default. Below there are three contextual factors, which make the overcoming of the continuum of violence related to the Ukrainian refugees in Bulgaria even more challenging.

### 4.1 Institutional “neglect” of GBV violence

Bulgaria is one of five EU member states that have refused to ratify the Council of Europe Convention on Preventing and Combating Violence against Women and Domestic Violence, commonly known as the Istanbul Convention (CETS No. 210, 2025). As a result of contentious political debates surrounding the Convention, some legislative amendments were introduced that expanded the criminalization of certain aspects of domestic violence. However, these changes also reinforced patriarchal norms by explicitly codifying restrictive definitions of “marriage” and “intimate relationships,” effectively anchoring women to a reproductive role and reinforcing dominant heteronormative frameworks (Roseneil and Stoilova, 2016).

A recent study on the barriers to identifying and prosecuting intimate partner violence in Bulgaria highlights a prevailing mistrust in the justice system, rooted in structural discrimination. This includes bureaucratic obstacles and a professional culture of inaction, particularly among male police officers (Pamporov, 2024a). Although Bulgaria is an EU member state, the institutional response to gender-based violence shares similarities with contexts outside the EU in which intersecting factors contribute to inadequate protections (Krause, 2015), and with patterns of institutional violence against asylum seekers in some EU countries (Sahraoui and Freedman, 2022).

Drawing from direct experience in training over 100 Bulgarian prosecutors, I can attest that a form of “soft misogyny” pervades the prosecutorial system. Misogynistic jokes, gendered stereotyping, and the underestimation of women’s professional competence are normalized elements of workplace culture—internalized even by some female prosecutors. The extent of institutional neglect is exemplified by the practice of the Office of the Cassation Prosecution, which acts as the national rapporteur on trafficking in human beings but fails to record any foreign victims of trafficking. The official justification—that foreign nationals lack Bulgarian Personal Identification Numbers (PINs) and are thus excluded from PIN-based databases—effectively renders all foreign victims invisible (as stated by a female prosecutor during a public debate with the present author).

Consequently, Bulgaria continues to report 100% of identified trafficking victims as Bulgarian nationals, reinforcing its official image solely as a source or transit country, not a destination. Yet evidence of the sexual exploitation of Ukrainian women in Bulgaria has existed since at least 2016, although these cases remain officially undocumented and absent from national statistics. Referring back to the framework proposed by Manjoo (2012), this pattern of institutional failure qualifies as violence perpetrated or condoned by the state—encompassing the failure of public institutions and law enforcement to prevent, investigate, or punish acts of gender-based violence. This climate of impunity impacts all women and girls residing in Bulgaria, but disproportionately affects displaced Ukrainian women and girls, whose compounded vulnerability is shaped by intersecting layers of gender, migration status, and nationality.

### 4.2 The national stereotypes

It is well established in feminist scholarship that misogyny and xenophobia often intersect and reinforce one another (Cooper, 2016). Accordingly, it is not surprising that national stereotypes and prejudices constitute a second dimension of the continuum of violence experienced by Ukrainian refugee women in Bulgaria. Although Slavic peoples are frequently described as “brothers” in cultural and historical narratives, a distinctly macho culture among Bulgarian men constructs women from countries such as the Czech Republic, Poland, Russia, and Ukraine as sexually permissive or hyperfeminized. These gendered and nationalized stereotypes are deeply normalized, often reproduced through misogynistic anecdotes that date back to Soviet-era tourism, including the figure of the Bulgarian seaside “seagull”—a local man associated with casual sexual encounters and the performative masculinity of so-called “flirty fishing.”

Unfortunately, certain individual media cases and controversial public figures of Slavic origin further fuel these myths.^12^ Such dynamics illustrate how stereotypes operate: they not only seek confirmation but actively construct their own apparent validation (Merton, 1948). In the case of Ukrainians, these processes are exacerbated by an additional layer of stigma—a widespread belief that Ukrainians are merely “more criminal Russians,” with little perceived cultural or national distinction from citizens of the Russian Federation. As a result, social distance toward Ukrainians tends to be greater than that toward Russians in Bulgaria (Pamporov, 2009). Echoing observations about interethnic relations in post-Yugoslav contexts (Morokvasic-Müller, 2004), many Bulgarians do not recognize the distinctiveness of post-Soviet Slavic-speaking populations. Instead, a Belarusian, Ukrainian, or even Kazakh or Komi woman married to a Bulgarian man is routinely referred to as a “Russian bride.”

### 4.3 Cultural myths related to sexual violence

The stereotype of women as “sexually available” is rooted not only in anecdotal tourist experiences from the communist era but also in deeply embedded social myths about rape and sexual violence in Bulgaria. A nationally representative randomized survey conducted in 2018 revealed a high degree of normalization of violence against women and girls, particularly through the widespread acceptance of rape myths and myths surrounding child sexual abuse. Alarmingly, the study found a significant lack of awareness about consent and sexual violence, with even survivors internalizing these harmful beliefs. A notable proportion of respondents expressed agreement with statements such as “If a woman does not resist, it cannot be considered rape” (Pamporov, 2024b). These social myths, normalizing sexual assault, serve as a powerful example of the continuum of violence and the internalization of misogyny (Leidig, 1992).

In this context, the self-reported increase in verbal abuse and public expressions of hatred experienced by Ukrainian refugee women in Bulgaria (UNHCR, 2025) should be understood as part of this broader continuum and omit regarded as a serious warning sign for the need for community-based prevention. To uphold the ethical principle of primum non nocere in applied social research, this article does not disclose the specific locations of accommodation facilities identified as being linked to commercial sex services. However, areas in cities such as Varna, Burgas, and Plovdiv have been locally recognized for such associations even prior to the arrival of Ukrainian refugees. The state accommodation offered to displaced women in these areas further reinforced pre-existing cultural myths—such as “they came for that,” “they want that,” “they are looking for that,” and “they are easy.” Today, these narratives target Ukrainian women and girls, but tomorrow, they could easily be applied to any female individual relocated to these stigmatized environments.

## 5 Conclusion and limitations of the current study

This article draws upon qualitative data and secondary analysis of six previous studies, which primarily addressed the arrival, accommodation, and access to healthcare and education for Ukrainian refugees in Bulgaria. As such, the proposed typology of refugee “waves” may contain omissions or interpretive biases. Still, the emergent patterns offer critical insight into the gendered and temporal dynamics of displacement. In response to ongoing gaps in knowledge, the Bulgarian office of UNHCR commissioned a targeted survey on gender-based violence (GBV) in late 2024, entrusted to the Demetra Association. At the time of writing, the project has reached the stage of culturally adapting interview guides, though political instability and UN budget constraints (linked to U. S. funding withdrawals) have delayed fieldwork.

Despite these limitations, this study reveals that the experiences of Ukrainian refugee women in Bulgaria are structured by a multidimensional continuum of violence, operating along four intersecting axes:

### 5.1 A continuum from structural to interpersonal violence

From policy decisions and legal statuses to everyday interactions, violence unfolds across scales. For instance, the Bulgarian Council of Ministers’ May 2025 reform introduced a housing scheme that ties continued shelter to market-based rents, offering only a minimal, short-term subsidy.^13^ This abrupt change exemplifies what Tastsoglou (2022) and others describe as slow violence—an incremental erosion of rights that appears bureaucratic but produces deep material insecurity. That same policy shift illustrates the “violence of uncertainty” (Grace et al., 2018; Grace et al., 2025), particularly for women without stable income or legal literacy. As seen in all waves, these macro-level decisions often translate into interpersonal violence, including economic coercion, survival sex, and sexual harassment.

### 5.2 A cyclical continuum between precarity and GBV

The recursive relationship between socio-economic precarity and exposure to GBV is well documented by the international scholarship (Kiss et al., 2012; Di Matteo, 2022; Reilly et al., 2022). It is observed also in the current study on the Ukrainian refugees in Bulgaria. Women who are legally liminal or economically dependent are at higher risk of being victimized, while experiences of GBV, in turn, intensify economic marginalization and psychological vulnerability. Among the “zero wave” (2014–2021), for example, women were often invisible to institutions and experienced the protracted harms of asylum limbo, cultural stigma, and domestic violence. One case—directly observed by me in 2017—when a victim of severe domestic abuse declined to testify out of “fear of deportation” illustrates how precarity silenced some refugee survivors—a contrast to Bulgarian women, who, while also affected by systemic neglect (Ivanova, 2016), do not face the additional threat of expulsion.

### 5.3 A spatial and temporal continuum of violence

The wave-based typology—zero, first, second, third, and fourth—reveals how different kinds of violence manifest across time and place. The “zero wave” faced prolonged asylum processes and cultural misrecognition, shaped by stereotypes about “Russian-speaking Ukrainians” (Pamporov, 2009). The “first wave” (February–April 2022) entered a space of legal liminality due to the implementation of EU Council Directive 2001/55/EC, which replaced traditional asylum with temporary protection, creating confusion and bureaucratic fragmentation. The “second wave” (April–June 2022) was isolated in coastal tourist hotels, where integration became practically impossible and moreover, increased nation-based prejudices. The “third wave” (June 2022 onward) included women with transnational family ties and greater institutional awareness—yet they still encountered structural misogyny and a lack of GBV support systems. Finally, the “fourth wave” embodies the concept of “pulsing diasporas” (Pamporov, 2024c), with women commuting physically and emotionally between Ukraine and Bulgaria. One 36-year-old participant captured this dual alienation:

*“We are already foreigners here and there. When we stay here, some people say: ‘Why do not you go there and fight for your country?!’ When we are there, some acquaintances say: ‘You know nothing about here. You abandoned us for your safety. So go back there!’”*

### 5.4 A continuum of normalized and invisible violence

Building on Kelly’s (1988) foundational concept and its recent extensions by Sisic et al. (2024) in refugee contexts, this final dimension emphasizes how institutional inaction, bureaucratic limbo, and cultural stereotypes normalize violence against refugee women and what the specific and unique forms of violence against refugee women may be. These forms of harm are often not even recognized as violence—despite having tangible, long-term effects. Slow violence, as theorized by Nixon (2011, 2013) and applied to border regimes by Schindel (2019), captures the insidious, cumulative impact of policies that erode protections without overt brutality. These harms rarely provoke immediate crisis, but they compound over time, trapping women in cycles of legal, economic, and emotional vulnerability. Unfortunately, Bulgaria in that respect seems not a hospitable host for women seeking international protection, because Bulgarian women also suffer from the same normalized violence, and obviously there is a long way that Bulgarian society has to go.

In summary, the experiences of Ukrainian refugee women in Bulgaria are shaped by a multidimensional continuum of violence that cuts across structural policies, interpersonal dynamics, spatial locations, and temporal moments. Each wave highlights distinct but overlapping vulnerabilities, from deportability and dependency to institutional neglect and transnational strain. These women are subject to multiple layers of gender-based violence—domestic, community, institutional, and transnational—while struggling to transcend cultural myths, national stereotypes, and the slow violence of institutional neglect. Addressing their needs requires not only humanitarian protection but also feminist-informed, intersectional, and structurally responsive policy solutions that move beyond symbolic inclusion toward genuine safety and justice.

## Data Availability

The datasets presented in this study can be found in online repositories. The names of the repository/repositories and accession number(s) can be found at: https://microdata.unhcr.org/.

## Footnotes

^1^For the sake of the article and in line with the UN policy, as well as following the Convention relating to the status of refugees (Article 1, paragraph 2), the Ukrainian citizens with temporary protection status will be labeled as Ukrainian refugees, despite the discrepancy between that and the current Bulgarin legal definition.

^2^The data is available through Ukrainian situation webpage of UNHCR, URL: https://data.unhcr.org/en/dataviz/311?sv=54&geo=10816 (last visited: 30.05.2025).

^3^Pomorie is a coastal small town and SPA resort (approximately 15,000) in Burgas province, known for his “Little Moskow” neighborhood with diaspora of approximately 3,000 permanent living Russian immigrants and with over 8,000 properties, owned by Russian citizens.

^4^The Blue Dots were support hubs, established in partnership with local authorities and partners including UNHCR to provide integrated free of charge services for refugee children and families fleeing the war in Ukraine. More information about is available at the UNHCR web page at URL: https://data.unhcr.org/en/documents/download/91739 (last visited on 12.04.2025).

^5^There is no academic research on the topic yet, but a brief content analysis of the press in May 2022, as well as some statements of leading populist politicians (both left and right spectrum), easily shows that.

^6^The data about frequency and duration of the air alarms in Ukraine is available at URL: https://air-alarms.in.ua/en

^7^*Kuritsa nye ptitsa, Bolgaria nye zagranitsa* (Rus.: Курица не птица, Болгария не заграница)—a Soviet time proverb that comes to express the feeling of being welcome in Bulgaria with regard of the Russian speaking USSR citizens due to similar Cyrillic alphabet use and “bratushki” (i.e., “brothers”) attitude towards the Eastern Slavs: Russians, Belarusians and Ukrainians.

^8^The Golden sands (in Varna region) and Sunny beach (in Burgas region) are famous Bulgarian seaside resorts, built in the 1960s and very well known in the post-USSR countries, which enormously enlarged in the recent years. The current tourist capacity of Golden sands is approximately 32,000 beds in 78 hotels, and the capacity of the Sunny beach is over 42,000 beds in 117 hotels.

^9^The state grant was set as 40 BGN per day per person (approximately 20 EUR).

^10^After the Crimean war (1853–1856), many Bulgarian refugees escaped the Ottoman empire and settled in the then Russian empire, in regions known as Bessarabia (nowadays divided between Ukraine and Moldova) and Tavria (a territory nowadays split in three Ukrainian regions Zaporozhia, Kherson and Crimea). According to the Ukrainian census in 2001, there were 204,574 ethnic Bulgarians in Ukraine, living mainly in those regions.

^11^There are two types of PIN in Bulgaria: UCN (Unified citizen number) and PNF (personal number of the foreigner). The latter is received by the refugees if they keep their citizenship of origin. It is used for taxation and social security purposes.

^12^For example, Natalia Kobilkina—a sexologist of Russian origin, published a controversial book “69 sex advices” and offer couching sessions labeled “Being geisha is a business.” It is the Bulgarian “fempreneur” analogue of Kim Karadarshian or Anna Bey.

^13^A one-time housing subsidy equivalent to 60 days of accommodation and capped at BGN 19.72 per day (approximately EUR 10 or USD 11.40 as of May 30, 2025).
